# Host–Guest Allosteric Control of an Artificial Phosphatase

**DOI:** 10.1021/jacs.9b12699

**Published:** 2020-03-26

**Authors:** Joanna Czescik, Yanchao Lyu, Samuele Neuberg, Paolo Scrimin, Fabrizio Mancin

**Affiliations:** Università di Padova, Dipartimento di Scienze Chimiche, via Marzolo 1, 35131 Padova, Italy

## Abstract

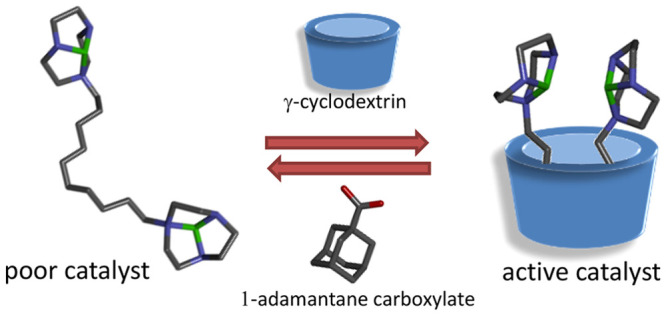

The activity of many enzymes is regulated by associative processes. To model this mechanism, we report here that the conformation of an unstructured bimetallic Zn(II) complex can be controlled by its inclusion in the cavity of a γ-cyclodextrin. This results in the formation of a catalytic bimetallic site for the hydrolytic cleavage of the RNA model substrate HPNP, whose reactivity is 30-fold larger with respect to the unstructured complex. Competitive inhibition with 1-adamantanecarboxylate displaces the metal complex from the cyclodextrin decreasing the reactivity.

Allosteric enzymes provide Nature with the possibility of regulating the rate of transformation of selected substrates,^1^ granting a powerful tool for biological information processing and signal transmission. In most allosteric enzymes, a small molecule effector/modulator binds to a regulatory site far from the catalytic site, causing a change of the enzyme’s conformation that modulates the catalytic activity.^2^ However, other regulatory mechanisms are used to trigger such conformational changes. These include chemical transformations (phosphorylation, cleavage) and the interaction/association with other proteins or membranes.^3^

Mimicking this behavior has attracted considerable interest over the years.^4,5^ In most of the examples reported to date, the activity of artificial catalysts is triggered by small molecule effectors, in particular metal ions.^4,6^ These provide strong and directional interactions that allow the conformation of suitable pro-catalysts to be controlled. A related strategy, proposed by Mirkin and co-workers,^7^ uses metal ions as structural elements and takes advantage of the different coordination geometries they can assume. The binding of small molecules to these metal centers changes their preferred coordination geometry and consequently the conformation of the catalyst. To the best of our knowledge, examples where the catalyst conformation is controlled by a supramolecular interaction with a molecule of similar size, resembling the mechanism of enzyme modulation by protein–protein interaction, have not been reported.

Zn(II) complexes of 1,4,7-triazacyclonane (TACN) are known to accelerate the hydrolytic cleavage of phosphate diesters, as the RNA model substrate HPNP (Chart 1), mainly by acting as Lewis acids.^8^ The activity is moderate, but it can be substantially increased by arranging two or more metal ions at a distance of about 4–6 Å (Chart 1). This organization can be achieved by using rigid scaffolds or by clustering several TACN·Zn(II) complexes on the surface of nanoparticles or in surfactant aggregates (see Chart 2).^8^ The controlled variation of the intermetallic distance, due to metal binding or photochemical switching, has been used to obtain allosteric control of the hydrolytic activity.^4^ Our goal was to investigate whether the intermetallic distance could be controlled by interaction with a suitable host, capable of binding an unstructured bimetallic TACN·Zn(II) complex inducing its folding to a catalytically active conformation.

**Chart 1:**
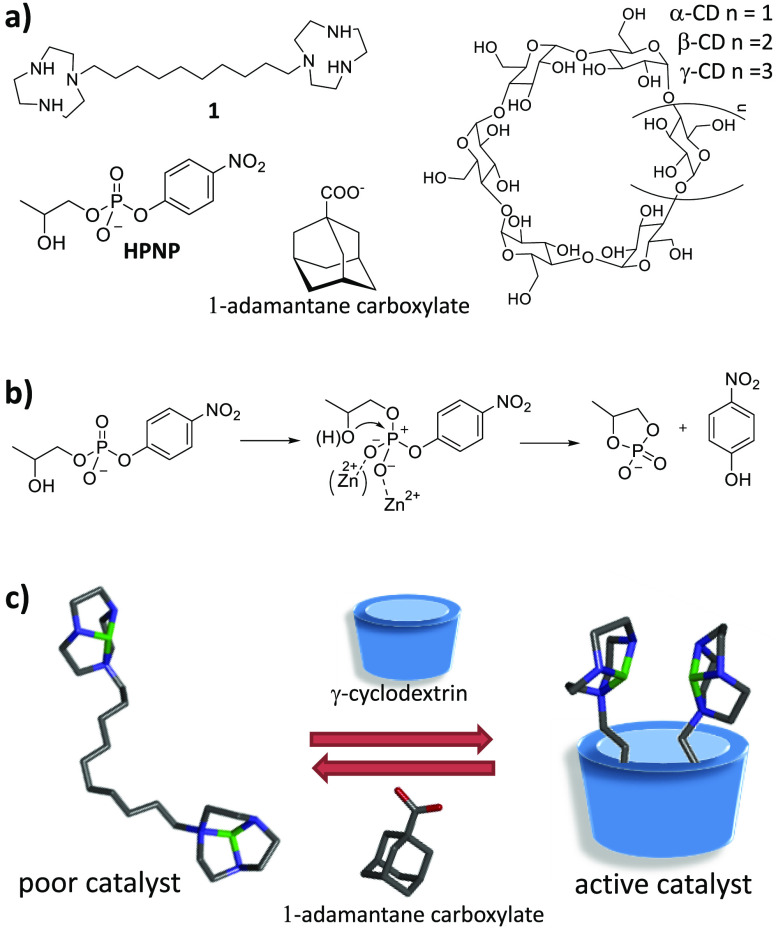
(a) Ligand **1** and Other Compounds Used in This Work; (b) General Mechanism for the HPNP Transesterification Catalyzed by Mono- and Dimetallic Zn(II) Complexes;^8a^ (c) Proposed Mechanism for the Allosteric Modulation of the Activity of Catalyst Studied (The Structure Drawn Are Molecular Models with Hydrogens Omitted: Gray, C; Blue, N; Red, O; Green, Zn^2+^)

**Chart 2:**
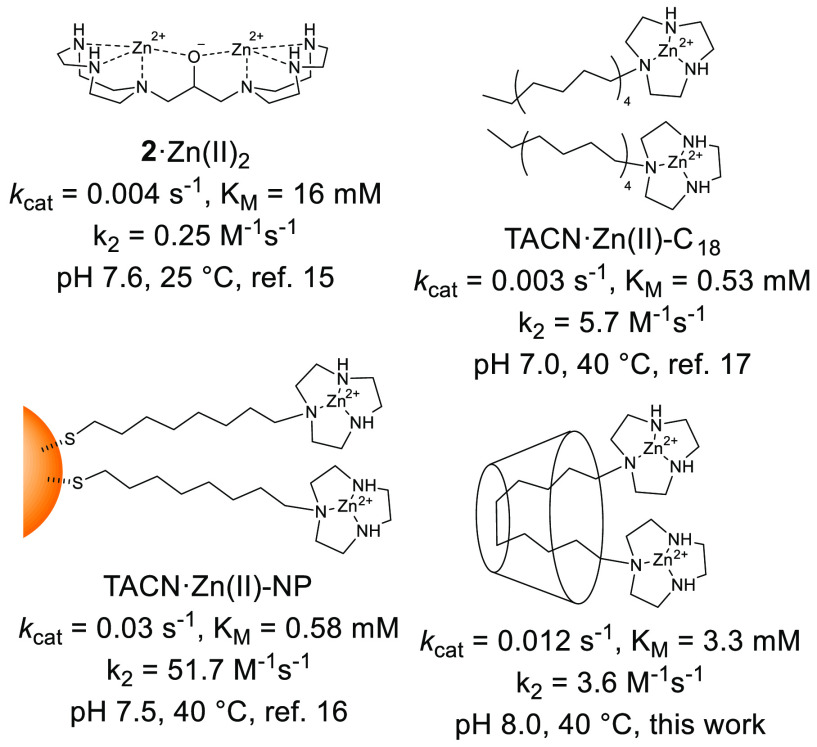
Structure and Reactivity Parameters for Different HPNP Cleaving Bimetallic Systems Reported in Literature or Studied in This Work

It is known from J. Rebek and co-workers that cavitands can host bifunctional linear alkanes in water forcing them to assume a U conformation that favors cyclization reactions.^9^ Inspired by this example, we designed and synthesized ligand **1** (Chart 1) where two TACN units were connected by a flexible and hydrophobic C_10_ alkyl spacer that could act as binding site for suitable receptors. Addition of Zn(NO_3_)_2_ to a water solution of **1** resulted in the formation of the corresponding bimetallic complex in situ. HPNP transesterification in the presence of **1**·Zn(II)_2_ was investigated by monitoring the absorbance increase of the released *p*-nitrophenol at 400 nm. Reaction rates were measured with the initial rates method (details in the Supporting Information (SI)).

As expected, **1**·Zn(II)_2_ is quite a poor catalyst. In water at pH 8.0 and 40 °C, the rate of HPNP cleavage increased linearly with the complex concentration in the concentration interval studied (0.1–0.5 mM, Figure 1A).^10^ From these data, a second-order rate constant of 0.15 ± 0.004 M^–1^ s^–1^ could be calculated. Taking into account that each **1**·Zn(II)_2_ complex bears two TACN·Zn(II) centers, this value is 3-fold larger than that (0.022 M^–1^ s^–1^) reported for the TACN·Zn(II) catalyzed reaction,^11^ suggesting that the two metal centers in **1**·Zn(II)_2_ act in a quasi-independent way. The behavior observed is fully consistent with that of similar bimetallic complexes which lack structural elements capable of holding the two metal ions in close proximity.^12^

**Figure 1:**
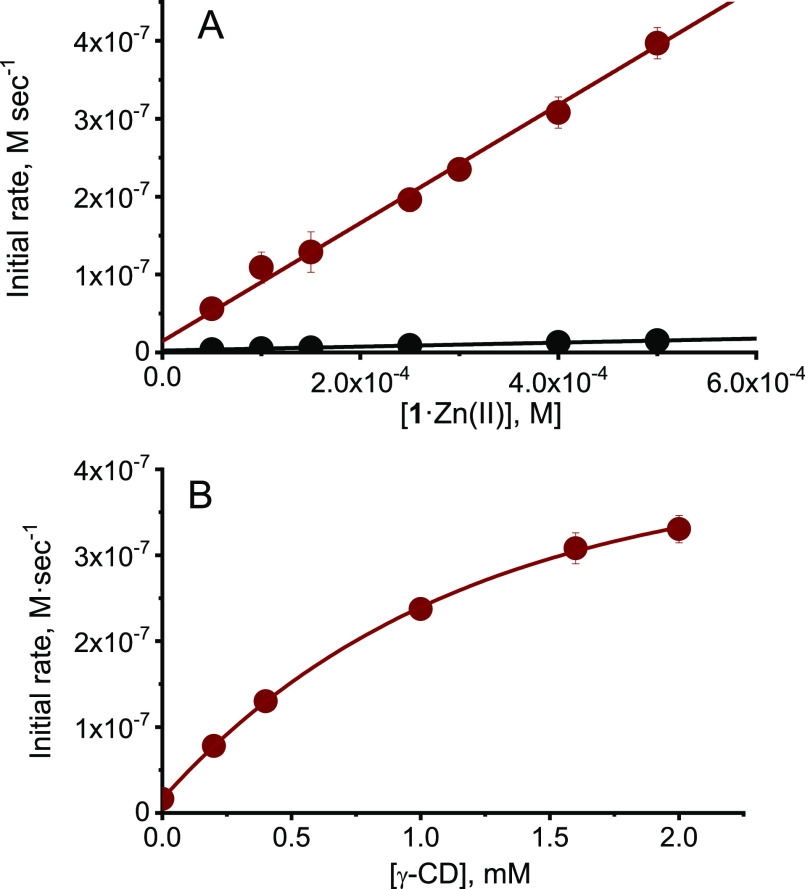
(A) Initial rates of HPNP cleavage as a function of **1**·Zn(II)2 concentration in the absence (black) or in the presence (red) of 5.0 mM γ-CD. (B) Initial rates of HPNP cleavage promoted by **1**·Zn(II)_2_ (0.5 mM) in the presence of increasing concentration of γ-CD. Conditions: pH = 8.0, [EPPS buffer] = 0.02 M, [HPNP] = 1.7 × 10^–4^ M, 40 °C. The lines represent the best fit of the experimental data.

Being granted the absence of an effective cooperation between the two metal ions in **1**·Zn(II)_2_, we proceeded to investigate whether we could obtain a cavitand-induced folding of the metal complex. The above experiment was hence repeated in the presence of a 5 mM concentration of γ-cyclodextrin (γ-CD). In this case, the rate of HPNP cleavage was found to be substantially larger (Figure 1A). Linear fitting of the kinetic data yielded a second-order rate constant of 4.5 ± 0.1 M^–1^ s^–1^. This figure corresponds to a 30-fold reactivity increase with respect to complex **1**·Zn(II)_2_ alone, and to a 210-fold reactivity increase with respect to TACN·Zn(II). The formation of a ternary complex between **1**·Zn(II)_2_ and γ-CD was confirmed with a different experiment, where the concentration of γ-CD was progressively increased by keeping the concentration of **1**·Zn(II)_2_ constant at 0.5 mM (Figure 1B). The rate of HPNP cleavage increased with the γ-CD concentration following a saturation profile. Fitting of these data with a 1:1 binding model provided an apparent binding constant of (1.0 ± 0.1) × 10^3^ M and a maximum rate of (5.1 ± 0.2) × 10^–7^ M s^–1^. This maximum rate corresponds to a 32-fold rate acceleration over the reaction of the sole **1**·Zn(II)_2_, in agreement with the previous experiment. Repeating the experiment in the absence of **1**·Zn(II)_2_ did not evidence any increase of the background reaction (Figure S11), confirming that γ-CD did not have any relevant independent activity in the conditions used. In addition, α- and β-cyclodextrins did not produce any effect on the rate of HPNP cleavage in the presence of **1**·Zn(II)_2_ (Figure S12). Hence, a relatively large host is required to enhance the reactivity of **1**·Zn(II)2.

The observation of a remarkably high reactivity of the binary system **1**·Zn(II)_2_/γ-CD does not grant per se the formation of a catalytic bimetallic site due to the folding of the metal complex. Evidence regarding the possible structure of the reactive species comes from a deeper kinetic investigation. First, we performed the kinetic version of the Job plot, where the reaction rate was measured at different **1**·Zn(II)_2_/γ-CD molar ratios while keeping the total concentration constant (Figure 2A). This experiment provided a bell-shaped profile with a maximum at 0.5 ratio, suggesting that in the reactive state **1**·Zn(II)_2_ and γ-CD are present in a 1:1 ratio. Subsequently, we measured the rate of HPNP cleavage at different concentrations of Zn(II) while keeping the concentrations of **1** and γ-CD constant (Figure 2B). A sigmoidal rate profile was obtained. Up to Zn(II)/**1** ratios smaller than 1 the cleavage rate increased only marginally by increasing the Zn(II) concentration. Then the reactivity steeply increased to eventually level off for Zn(II)/**1** ratios larger than 2. This behavior is usually considered strong evidence of a cooperative catalytic mechanism, where two metal ions interact simultaneously with the substrate providing a greater than additive stabilization of the increasing charge in the transition state.^13^ Clearly, the fraction of ligand **1** where both the TACN sites are occupied by a metal ion can be relevant only for Zn(II)/**1** ratios larger than 1.

**Figure 2:**
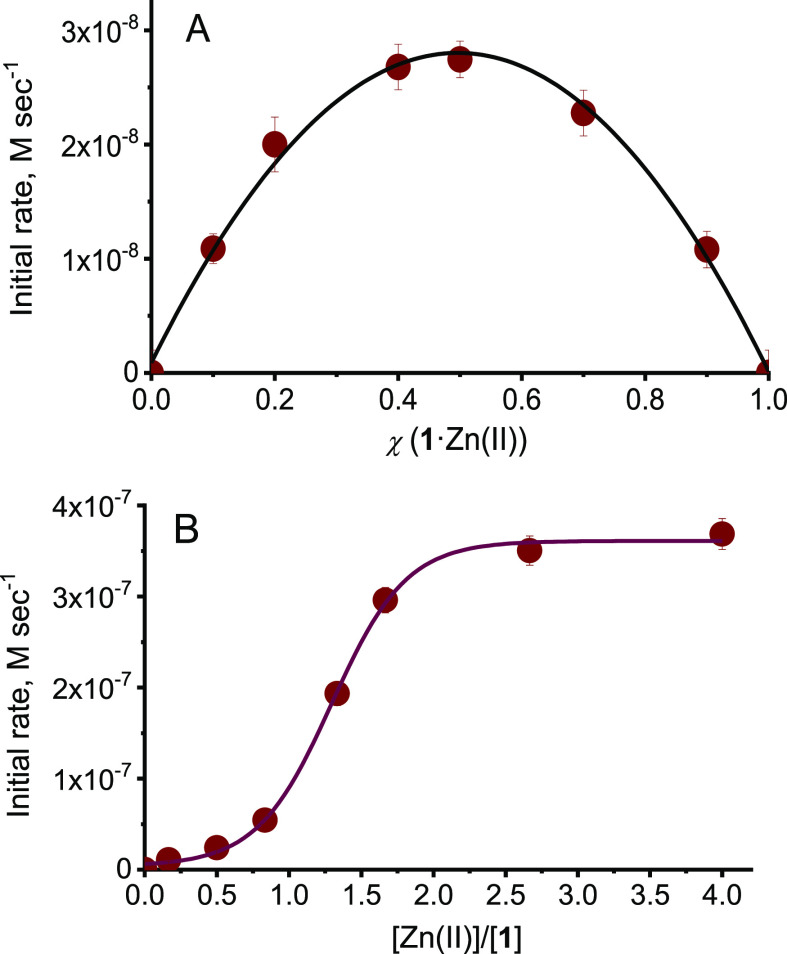
(A) Initial rates of HPNP cleavage as a function of the molar fraction of **1**·Zn(II)_2_ (corrected for the contribution of free **1**·Zn(II)_2_). The sum of the concentrations of **1**·Zn(II)_2_ and γ-CD was kept constant at 0.5 mM. (B) Initial rates of HPNP cleavage as a function of the [Zn(II)]/[**1**] ratio ([**1**] = 0.5 mM, [γ-CD] = 5 mM). Conditions: pH = 8.0, [EPPS buffer] = 0.02 M, [HPNP] = 1.7 × 10^–4^ M, 40 °C. The lines were drawn to help data interpretation.

Taken together the above experiments indicate that the reactive species is likely a 1:1 complex of **1**·Zn(II)_2_ and γ-CD. In this ternary complex, the two Zn(II) ions are held at a distance short enough to allow their simultaneous interaction with the substrate and the resulting cooperative catalysis. This evidence supports the mechanism depicted in Chart 1, where the inclusion of the hydrophobic linker in the cyclodextrin cavity forces the **1**·Zn(II)_2_ complex to assume a U-shaped conformation that brings in proximity the two ions. Additional NMR experiments confirmed the formation of an inclusion complex between **1**·Zn(II)_2_ and γ-CD. Indeed, only signals relative to the protons located inside the cyclodextrin cavity or on the rims underwent detectable changes of their chemical shift, as usually observed in the case of inclusion complexes (Figure S5). An NMR Job plot confirmed the 1:1 stoichiometry (Figure S8).^14^

Finally, the catalytic activity of the **1**·Zn(II)_2_/γ-CD was measured at increasing concentrations of HPNP. Concentrations of **1**·Zn(II)_2_ and γ-CD were fixed respectively at 0.15 and 1.5 mM. Fitting of the resulting saturation profiles with the Michaelis–Menten equation yielded a *k*_cat_ value of (0.012 ± 0.007) s^–1^ and a *K*_M_ value of (3.3 ± 0.4) mM. Chart 2 compares these values with the best-performing bimetallic TACN·Zn(II) hydrolytic catalysts reported in literature. These include the rigid **2**·Zn(II)_2_ studied by Morrow and Richards^15^ and considered to be optimally preorganized, the gold nanoparticles TACN·Zn(II)-NP studied by Scrimin,^16^ and the micelles TACN·Zn(II)-C_18_ studied by Prins and Chen.^17^ With the different reaction conditions taken into account, it appears that the reactivity of the **1**·Zn(II)_2_/γ-CD system is comparable to the other bimetallic species. In particular, the *k*_cat_ values reveal that the intrinsic reactivity of **1**·Zn(II)_2_/γ-CD is greater than that of the micellar system, similar if not better to that of **2**·Zn(II)_2_, and only slightly smaller than that of the nanoparticle system. The main advantages that **1**·Zn(II)_2_/γ-CD share with both the micellar and nanoparticle systems, with respect to **2**·Zn(II)_2_, are the greater positive charge of the catalytic site, due to the absence of the alkoxide bridge, and the flexibility. The first ensures greater electrostatic stabilization of the negatively charged transition state.^18^ The second allows the metal centers to adjust their position to match the needs of the reactive species formed during the reaction.^8b^ Interestingly, partially restrained systems as nanoparticles and **1**·Zn(II)_2_/γ-CD perform better than fully flexible ones as micelles. Analysis of the *K*_M_ values reveals that the affinity for HPNP of both micelles and nanoparticles is greater than that of **1**·Zn(II)_2_/γ-CD and **2**·Zn(II)_2_. This is likely due to the binding of the nitrophenyl moiety to the hydrophobic pseudophase formed by the micellar aggregate or by the monolayer. Such an effect is not present in **1**·Zn(II)_2_/γ-CD and **2**·Zn(II)_2_. Indeed, *K*_M_ values in the low mM range are typical for dinuclear Zn(II) complexes.^8b^ The greater affinity for the substrate of **1**·Zn(II)_2_/γ-CD with respect to **2**·Zn(II)_2_ can be again ascribed to the greater positive charge.

If the reactivity of the **1**·Zn(II)_2_/γ-CD is due to the formation of an inclusion complex, the catalytic activity of the system might be regulated by a competitive host acting as antagonist. To test this hypothesis, we measured the rate of the HPNP cleavage in the presence of **1**·Zn(II)_2_/γ-CD and of increasing concentrations of 1-adamantanecarboxylate, which is water-soluble and a well-known guest for cyclodextrins. Results are reported in Figure 3B. As expected the reaction rate decreased as the concentration of the adamantane derivative increased. Both the sigmoidal shape of the inhibition profile^19^ and the obtained value of the inhibitor binding constant (*K*_i_ = 4.6 × 10^3^ M^–1^)^20^ point against the possibility that the inhibition observed could arise by the competitive binding of the carboxylate to the bimetallic center. This hypothesis is further ruled out by the observation that addition of sodium acetate at the same concentration of 1-adamantane-carboxylate did not produce any effect on the reaction rate (Figure 3B). In a later experiment, a reaction was started with only **1**·Zn(II)_2_ and HPNP in a cell. Subsequently, γ-CD (1 equiv), 1-adamantanecarboxylate (2 equiv), and again **1**·Zn(II)_2_ (1 equiv) were added after fixed time intervals. This sequence of additions produced the turning on, off, and on of the reactivity, as expected on the basis of the proposed mechanism.

**Figure 3:**
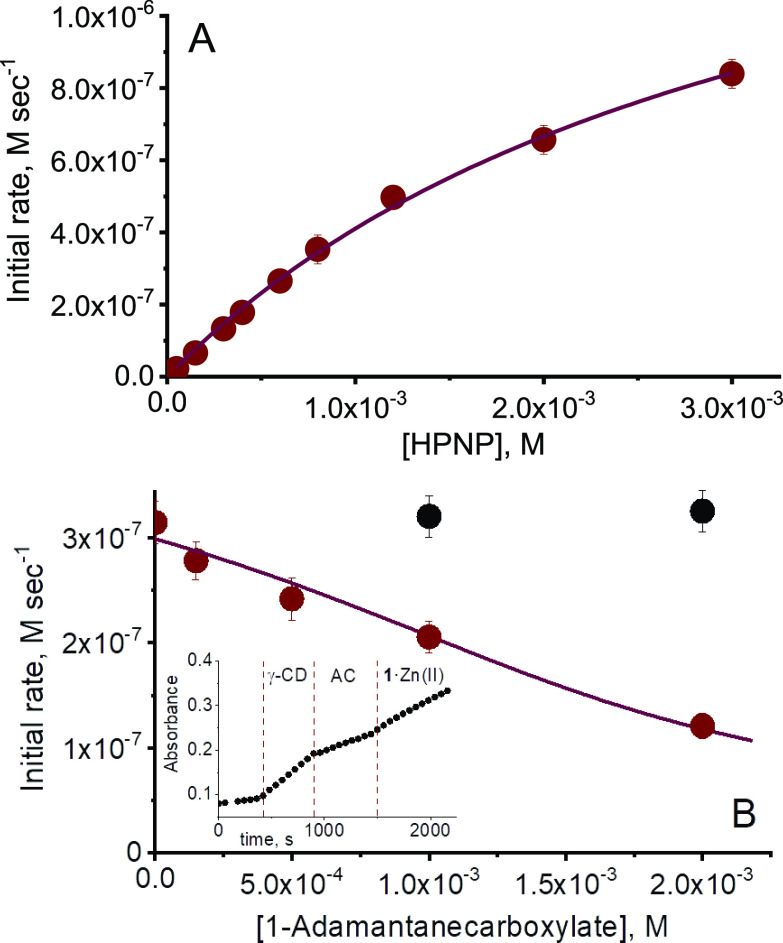
(A) Initial rates of HPNP cleavage in the presence of **1**·Zn(II)_2_ and γ-CD (1.5 × 10^–3^ M) at increasing HPNP concentration. (B) Initial rates of HPNP cleavage in the presence of **1**·Zn(II)_2_ and γ-CD (1.5 × 10^–3^ M) at increasing 1-adamantane-carboxylate (red) or acetate (black) concentration. *Inset:* kinetic trace obtained after subsequent additions of γ-CD, 1-adamantanecarboxylate, **1**·Zn(II)_2_. Conditions: [**1**·Zn(II)_2_] = 1.5 × 10^–4^ M, pH = 8.0, [EPPS buffer] = 0.02 M, [HPNP] = 5.0 × 10^–4^ M (in Figure 3B), 40 °C. The lines represent the best fit of the experimental data.

This study describes the first example of the activation of a catalyst by its inclusion into the cavity of a supramolecular host of comparable size. The mechanism proposed mimics the activation of catalytic sites by protein–protein association. In addition, it offers wide possibilities for the realization of complex chemical systems, where the reactivity is controlled by the balance of different components.

## Supporting Information Available

Synthesis and characterization of **1**, and additional kinetic experiments (PDF)
