# Supplementary material for: Host–Guest Allosteric Control of an Artificial Phosphatase
Source: J Am Chem Soc. 2020 Mar 26;142(15):6837–41. doi: 10.1021/jacs.9b12699 (PMC7997383; doi:10.1021/jacs.9b12699)
Supplement: Supplementary file 1 — ja9b12699_si_001.pdf [file ja9b12699_si_001.pdf]

# Supporting Information

## Host-guest allosteric control of an artificial phosphatase

Joanna Czescik, Yanchao Lyu, Samuele Neuberg, Paolo Scrimin and Fabrizio Mancin.

*Dipartimento di Science Chimiche and CNR-ITM, Università di Padova, via Marzolo 1, I-35131 Padova, Italy. Fax: +39 0498275239; Tel: +39 0498275666; E-mail: fabrizio.mancin@unipd.it*

Table of Contents

1. Experimental procedures ............ S2
2. Synthesis of ligand **1**............ S5
3. NMR experiments............ S9
4. Additional kinetic experiments............ S11

## 1. Experimental Procedures.

**General:** Solvents were purified by standard methods. All commercially available reagents and substrates were used as received. Water for kinetic experiments was purified with a Millipore MilliQ® apparatus (18 MΩ cm resitivity).

TLC analyses were performed using Merck 60 $F_{254}$ precoated silica gel glass plates. Column chromatography was carried out on Macherey-Nagel silica gel 60 (70-230 mesh).

NMR spectra were recorded using a Bruker AV III 500 spectrometer operating at 500 MHz for $^1$H, 125.8 MHz for $^{13}$C. Chemical shifts are reported relative to internal $Me_4Si$. Multiplicity is given as follow: s = singlet, d = doublet, t = triplet, q = quartet, qn = quintet, m = multiplet, br = broad peak.

ESI-MS mass spectra were obtained with an Agilent Technologies LC/MSD Trap SL mass spectrometer.

$Zn(NO_3)_2$ was an analytical grade product. Metal ion concentration in stock solutions were measured by atomic absorption spectroscopy using Perkin Elmer 1100 instrument. Buffers used for kinetic experiments were used as supplied by the manufacturers: 2-morpholinoethanesulfonic acid (MES, Fluka), 4-(2-hydroxyethyl)-1-piperazineethanesulfonic acid (HEPES, Sigma), 4-(2-hydroxyethyl)-1-piperazinepropanesulfonic acid (EPPS, Sigma), 2-[N-cyclohexylamino]ethanesulfonic acid (CHES, Sigma), ), 3-[cyclohexylamino]1-propanesulfonic acid (CAPS, Sigma).

The substrate 2-hydroxylpropyl 4-nitrophenyl phosphate (HPNP) was synthetized according to Brown's protocol.[1]

### Kinetic Measurements.

Stock solution of ligand **1**, cyclodextrins and HPNP were prepared in water. pH of the ligand's stock solution was adjusted to 6.5 with 1 M NaOH. Stock solution of acid 1-adamantanecarboxilic was prepared in EPPS buffer at pH 8.0.

Kinetic experiments were performed with Cary 50 spectrophotometer equipped with thermostatic multiple cell holders. HPNP conversion was monitored by the increase of the absorption at 400 nm of the *p*-nitrophenolate. Kinetic analysis was performed with the initial rates methods. Absorbance data relative to the first part of the reaction, corresponding to a substrate conversion smaller than 10-15% were collected. In these conditions, substrate concentrations can be assumed to be constant and equal to the initial concentrations. Initial rates of each run wre calculated by a linear fitting of the data collected. The slope values obtained from the fits were divided by the molar absorptivity (ε) of *p*-nitrophenol

[1] D. M. Brown, D. A. Usher, *Journal of the Chemical Society (Resumed)* **1965**, 6558-6564.

measured in the experimental conditions ($1.65\times10^5$ $cm^{-1}M^{-1}$) .. Experiments were performed in triplicate and data reported in the paper figures are the corresponding averages with standard deviations, which usually ranged between 5 and 10%.

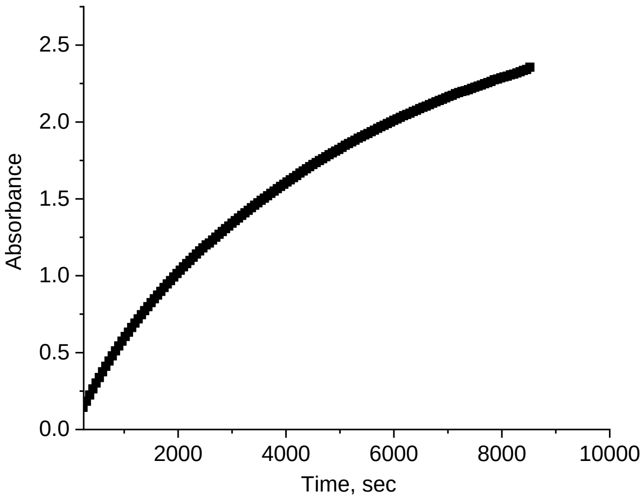


**Figure S1.** Kinetic profile obtained by prolonged incubation of HPNP in the presence of **1**·$Zn(II)_2$ at pH 8.0 and 40 °C. The almost complete cleavage of the substrate is observed (the expected absorbance increase for full cleavage is 2.8), demonstrating that **1**·$Zn(II)_2$ catalyzes the HPNP transesterification in agrrement with the mechanism proposed. Conditions: [**1**·$Zn(II)_2$] = $0.05 \times 10^{-4}$ M, [γ-CD] = $5 \times 10^{-3}$ M, [MES buffer] = 0.02 M, [HPNP] = $1.7 \times 10^{-4}$ M.

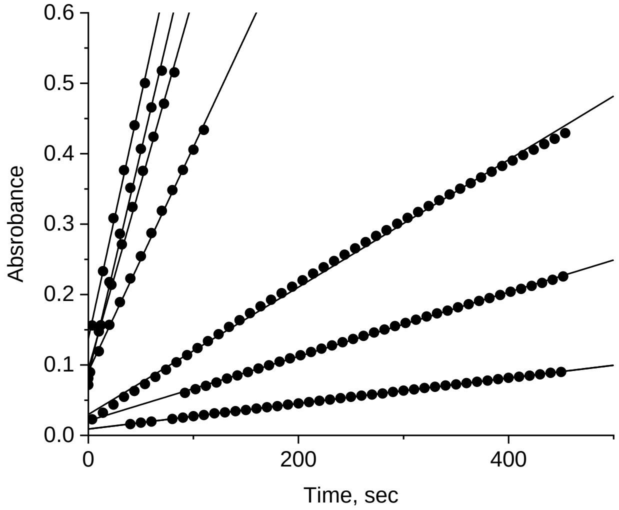


**Figure S2.** Row absorbance vs time data relative to one of the experiments performed to obtain the results reported in Figure 2B. Lines represent the linear fit of the data. Conditions: [**1**]= 0.5 mM, [Zn(II)] as reported in Figure 2B, pH = 8.0, [EPPS buffer] = 0.02 M, [HPNP] = $1.7 \times 10^{-4}$ M, 40 °C.

Linear fits and the fitting of the pH vs rate profile was performed with the OriginPro 2018 software package. The equation used to fit the pH profile was:

$$\text{rate} = \frac{v_{max}}{\frac{[\mathrm{H}^+]}{K_a} + 1}$$

where $K_a$ is the apparent $pK_a$ of the deprotonable species.

The fitting of the other kinetic data was performed using the DynaFit software package. The scripts used are reported here (0.nnn is the initial extimation of the parameter to be adjusted in the fitting).

For the experiments revealing binding equilibria (Fig. 1B, Fig. 2A)

```
[task]
  data = equilibria
  task = fit
[mechanism]
  E + S <==> ES    :   Ks     dissociation
[constants]
  Ks = 0.nnn ?
[concentrations]
  E = 0.0000                  (Concentration of the Zn(II) complex)
[responses]
  ES = 0.nnn ?                (Maximum rate)
[data]
  variable S                  (Cyclodextrin or HPNP)
  directory NAME
  sheet NAME.csv
  column 2
[output]
  directory NAME
[end]
```

For the experiments involving competitive equilibria (Fig. 2B), in this case data could not be fitted with the previous model:

```
[task]
  data = equilibria
```

```
   task = fit
[mechanism]
   E + S <==> ES     :    Ks      dissociation
   E + I <==> EI     :    Kd      dissociation
[constants]
   Ks = 0.001
   Kd = 0.nnn ?
[concentrations]
   E = 0.0015                     (Cyclodextrin)
   S = 0.00015                    (Concentration of the Zn(II) complex)
[responses]
   ES = 0.nnn   ?                 (Maximum rate)
[data]
   variable I                     (1-adamantanecarboxilate)
   directory NAME
   sheet NAME.csv
   column 2
[output]
   directory NAME
[end]
```

## 2. Synthesis of ligand 1

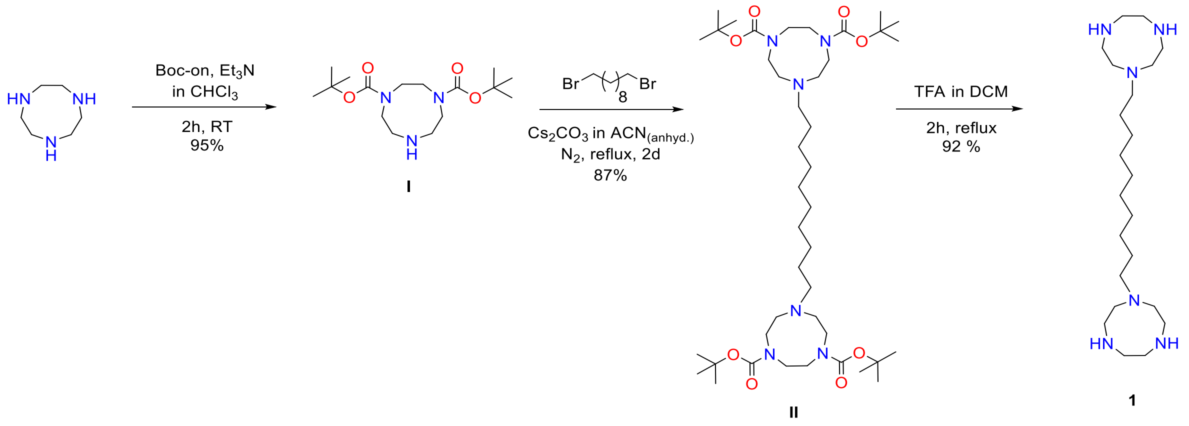


**Di-tert-butyl-1,4,7-triazonane-1,4-dicarboxylate (I):** In a round-bottom flask, 1,4,7-triazacyclononane (TACN; 1.0 g, 7.73 mmol, 1.0 eq.) and triethylamine (TEA; 2.3 g, 3.23 mL, 23.2 mmol, 3.0 eq.) were dissolved in 30 mL of chloroform and cooled in an ice bath. [2-(tert-butoxycarbonyloxyimino)-2-phenylacetonitrile] (Boc-on; 3.37 g, 15.5 mmol, 2.0 eq.) dissolved in 20 mL of chloroform was added dropwise over the course of 5 hours. After the addition was complete, the mixture was stirred at room temperature for additional 2 hours. Afterwards the solvent was evaporated and remaining yellow oily residue was extracted with 5% aqueous solution of sodium carbonate, brine and 10% aqueous solution of citric acid. The acidic aqueous phase was alkalized to pH 11 with 1 M sodium hydroxide and then extracted with chloroform. The organic solvent was evaporated and the pure product collected as a clear oil (95% yield).

*$^{1}H$ NMR ($CDCl_3$, 500 MHz), δ: 3.46-3.39 (m, 4H), 3.26-3.29 (m, 4H), 2.89 (m, 4H), 1.45 (s, 18H)*
*$^{13}C$ NMR ($CDCl_3$, 500 MHz), δ: 156.0, 155.8, 79.8, 79.7; 52.5, 52.4, 49.8, 49.5, 48.2, 48.09, 28.53*
*ESI-MS (m/z): 330.3 $[M+H]^+$*

**Tetra-tert-butyl 7,7'-(decane-1,10-diyl)bis(1,4,7-triazonane-1,4-dicarboxylate) (II)**: In a round-bottom flask previously purged with nitrogen, **I** (600 mg, 1.82 mmol, 3.0 eq.) and caesium carbonate (1.18 g, 3.64 mmol, 6.0 eq.) were suspended in 50 mL of anhydrous ACN. Then 1,10-dibromodecane (182 mg, 0.607 mmol, 1.0 eq) was added. The mixture was heated at 60˚C for two days under stirring. Afterwards, the solvent was removed and resulting crude was purified by column chromatography

(eluent dichloromethane/acetonitrile gradient from 95/5 to 70:30). The desired product was isolated as a translucent oil (87% yield).

*$^{1}H$ NMR ($CDCl_3$, 500 MHz), δ: 3.47-3.37 (m, 12H), 3.25-3.20 (m, 8H), 2.61-2.58 (m, 8H), 2.45 (m, 4H), 1.45-1.24 (m, 48H)*

*$^{13}C$ NMR ($CDCl_3$, 500 MHz), δ: 156.0, 155.9, 155.7, 79.29, 57.37, 57.21, 54.42, 54.00, 51.05, 50.97, 50.75, 50.57, 50.39, 50.11, 30.05, 28.92, 28.73, 28.44, 28.42, 28.30, 28.11, 27.93, 27.84, 27.78*

*ESI-MS (m/z): 797.7 $[M+H]^+$, 819.7 $[M+Na]^+$, 841.7 $[M+2Na]^+$*

**1,10-di(1,4,7-triazonan-1-yl)decane (1)**: In a two-neck round-bottom flask previously purged with nitrogen and placed in an ice bath, **II** (200 mg, 0.26 mmol, 1.0 eq.) was dissolved in 10 mL of dry dichloromethane. Trifluoroacetic acid (TFA; 0.2 mL, 2.6 mmol, 10 eq.) was dissolved in an equal amount of dichloromethane and added dropwise. Afterwards the reaction was stirred at room temperature for 2 hours. The solvent and TFA were evaporated and the remaining orange oil was washed several times with cold diethyl ether. Desired product was isolated as a TFA pale-yellow salt (92% yield).

*$^{1}H$ NMR ($CD_3OD$, 500 MHz), δ: 3.56-3.48 (m, 8H), 3.28-3.26 (m, 8H), 3.01-2.99 (m, 8H), 2.47-2.71 (m, 4H), 1.56-1.52 (m, 4H), 1.39-1.26 (m, 12H)*

*$^{13}C$ NMR ($CD_3OD$, 500 MHz), δ: 162.97, 162.69, 162.40, 162.12, 121.28, 118.96, 116.65, 114.33, 56.77, 44.41, 43.08, 30.50, 28.43, 25.74*

*ESI-MS (m/z): 397.4 $[M+H]^+$, 441.4 $[M+2Na-H]^+$*

*TOF ESI-HRMS: $[M-H]^+$ calcd. for $C_{22}H_{49}N_6$ = 397.4019. Found = 397.4025.*

*Elemental analysis* ($C_{34}H_{52}F_{18}N_6Na_2O_{12}$, **1** · 4 $CF_3COOH$ · 2 $CF_3COONa$)*: Called:* C: 36.31, H: 4.66, N: 7.47; *found*: C: 36.86, H: 4.25, N: 7.66

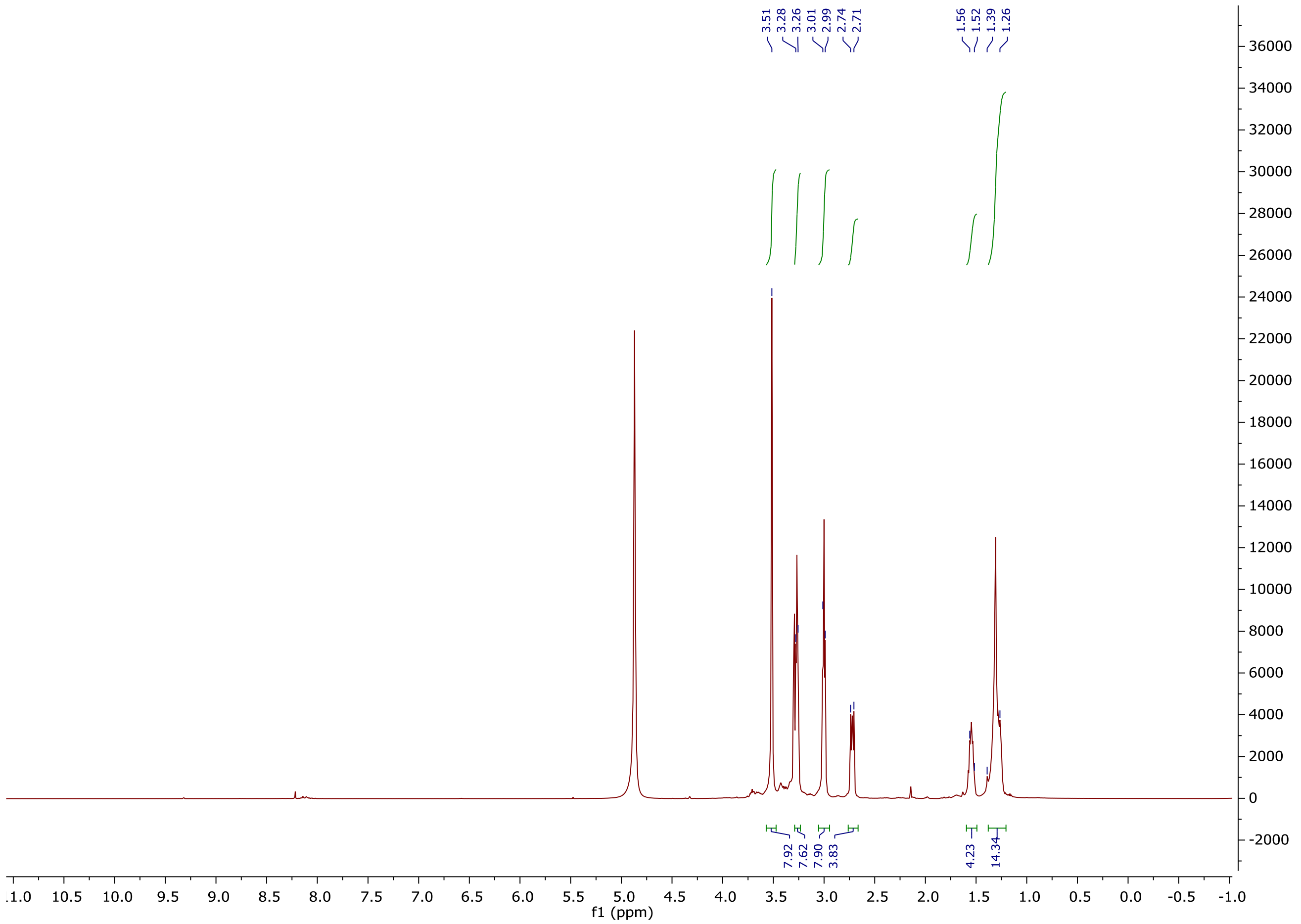


**Figure S3.** $^{1}$H NMR spectrum of the ligand **1** in $CD_3OD$.

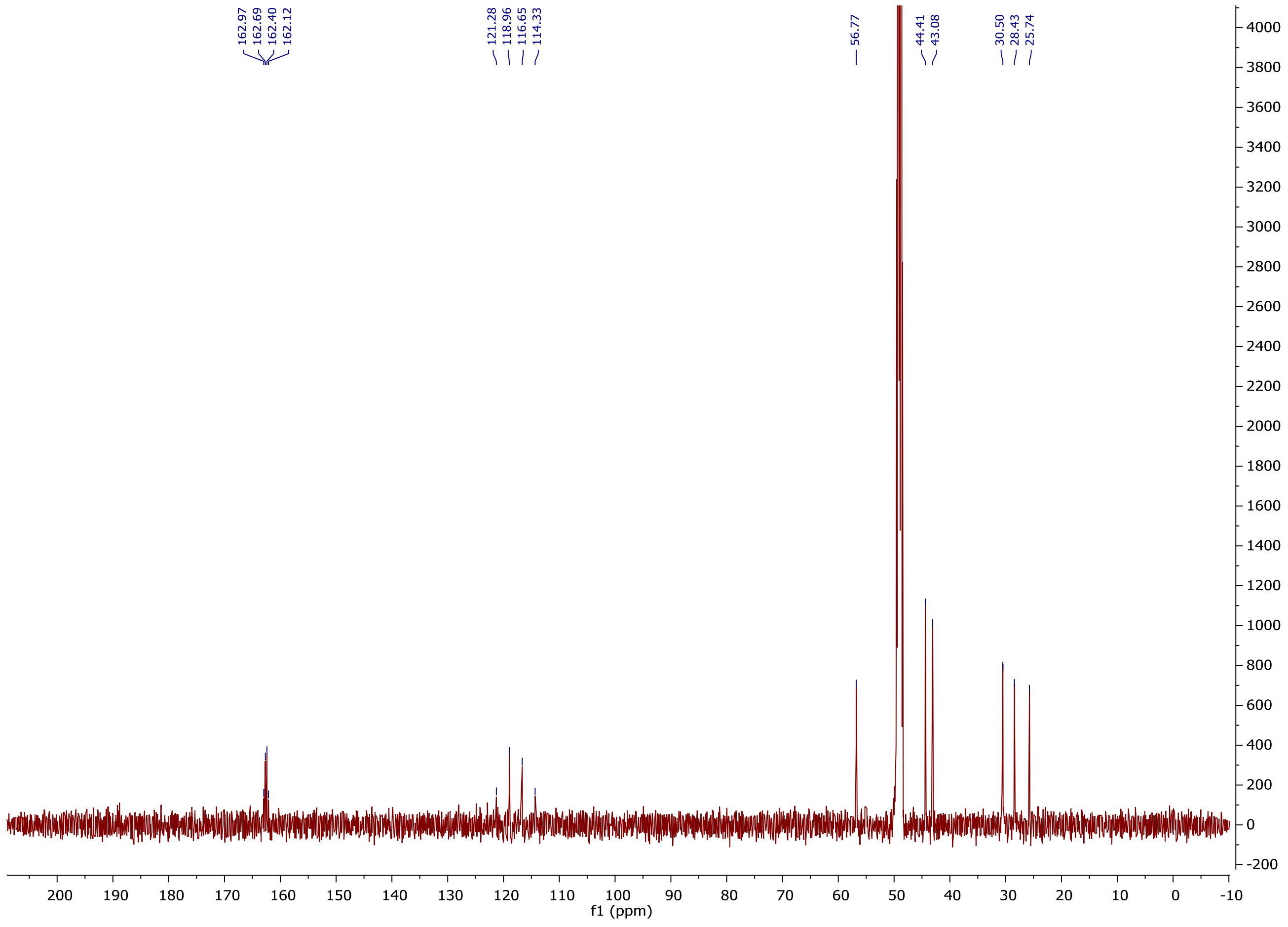


**Figure S4.** $^{13}C$ NMR spectrum of the ligand **1** in $CD_3OD$.

## 3. NMR experiments

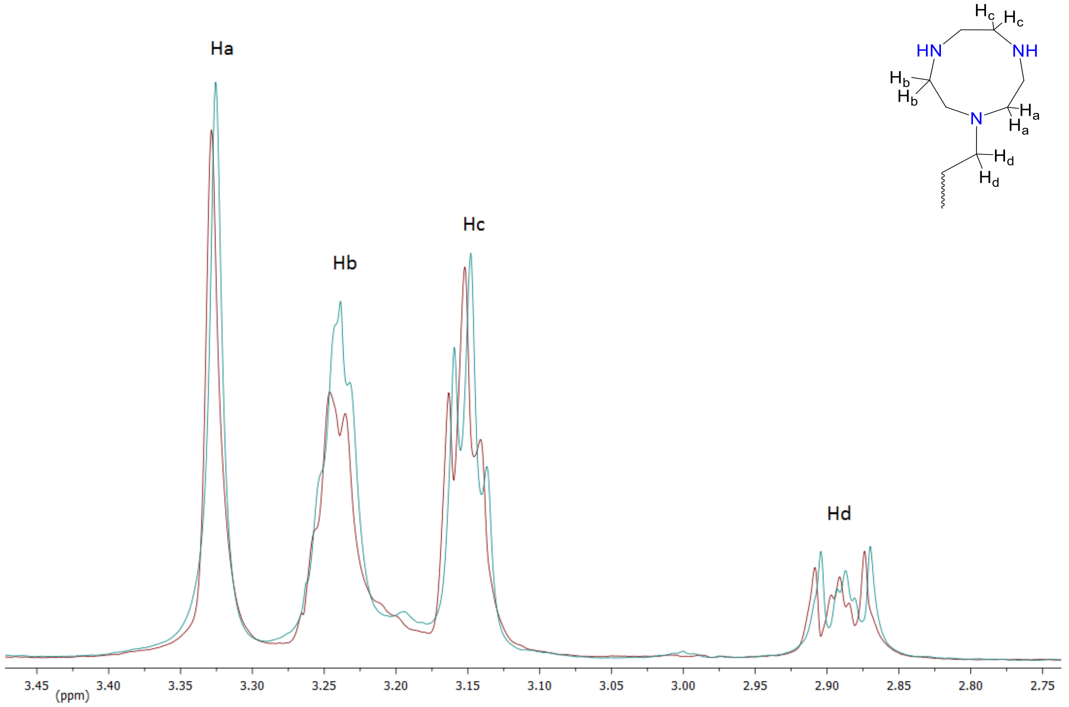


**Figure S5.** $^{1}$H NMR sub-spectra of the 1,4,7-triazacyclononane portion of the ligand **1** before and after the addition of γ-cyclodextrin. Conditions: [**1**·Zn(II)$_2$] = 0.5 mM, [γ-CD]= 7.5 mM in $D_2O$.

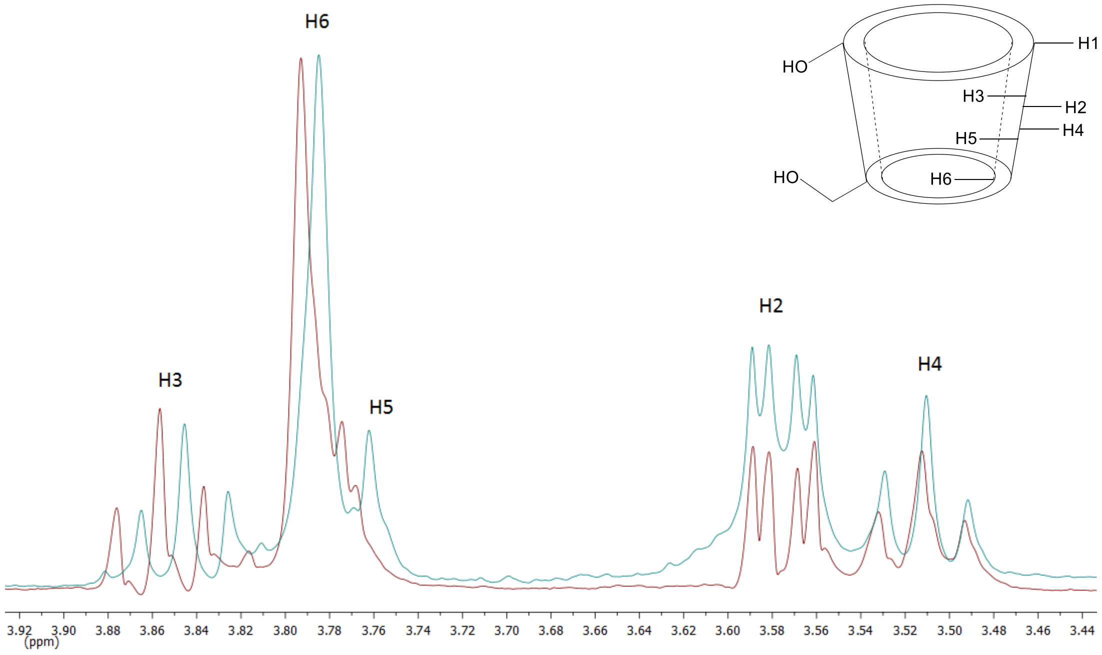


**Figure S6.** $^{1}$H NMR subspectra of the $H_2$-$H_6$ γ-cyclodextrin before and after the addition of the ligand **1**. Conditions: [**1**·Zn(II)$_2$ = 0.5 mM, [γ-CD]= 7.5 mM in $D_2O$. Relevant shift are observed only for inner $H_3$, $H_5$ and $H_6$ signals.

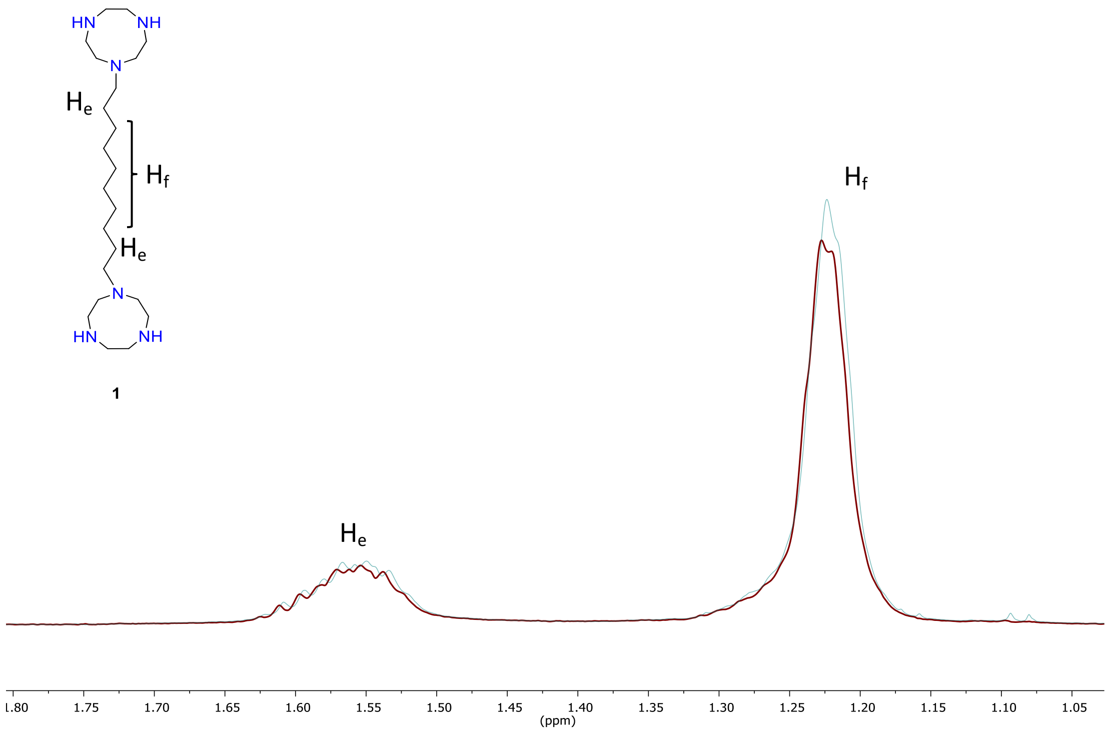


**Figure S7.** $^{1}$H NMR subspectra of alkyl the portion of the ligand **1** before and after the addition of γ-cyclodextrin. Conditions: [**1**·Zn(II)$_2$] = 0.5 mM, [γ-CD]= 7.5 mM in $D_2O$.

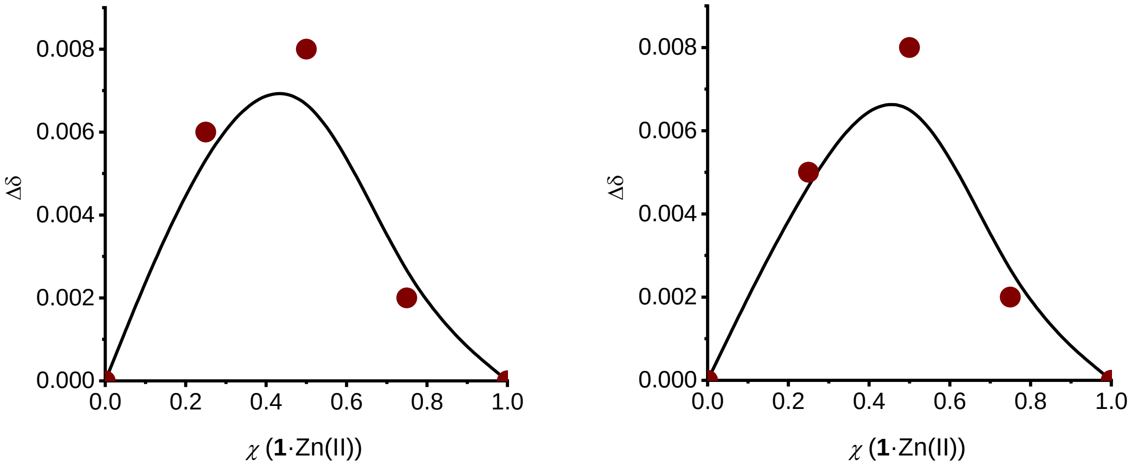


**Figure S8.** Chemical shift variation of the γ-CD H5 (left) and H3 (right) signals as a function of the molar fraction of **1**·Zn(II)$_2$. The sum of the concentrations of **1**·Zn(II)$_2$ and was kept constant at 0.5 mM in $D_2O$.

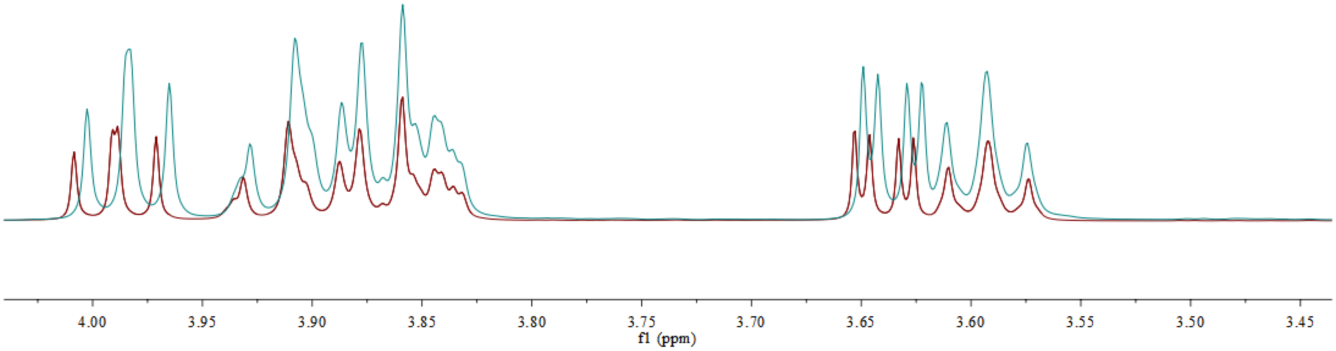


**Figure S9.** $^{1}$H NMR subspectra of the $H_2$-$H_6$ α-cyclodextrin before and after the addition of the ligand **1**. Conditions: [**1**·Zn(II)$_2$] = 0.5 mM, [γ-CD]= 7.5 mM in $D_2O$. Shift are observed for most of the signals. Interaction is between **1**·Zn(II)$_2$ appears to be unspecific.

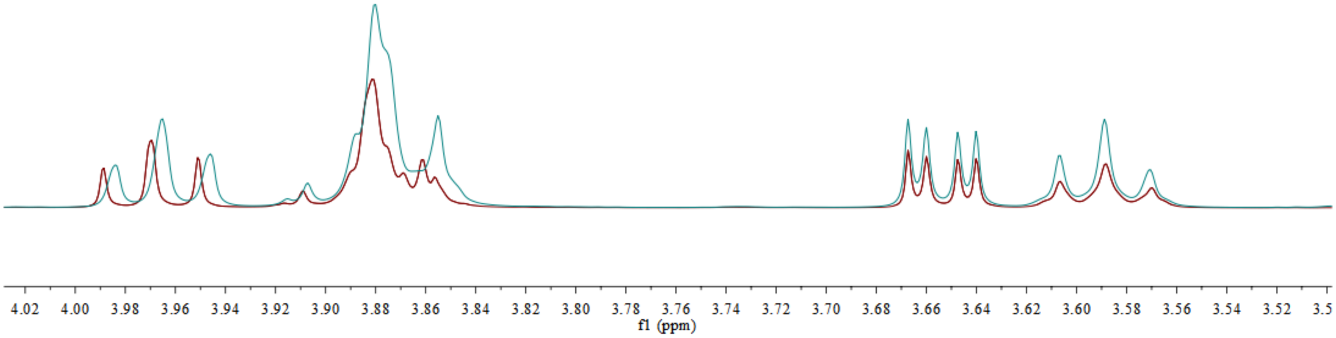


**Figure S10.** $^{1}$H NMR subspectra of the $H_2$-$H_6$ β-cyclodextrin before and after the addition of the ligand **1**. Conditions: [**1**·Zn(II)$_2$] = 0.5 mM, [γ-CD]= 7.5 mM in $D_2O$. Shift observed are similar to those obtained with γ-CD, in this case however the interaction is unable to fold the complex in a catalytically active conformation.

## 4. Additional kinetic experiments

### 4.1 Control experiments

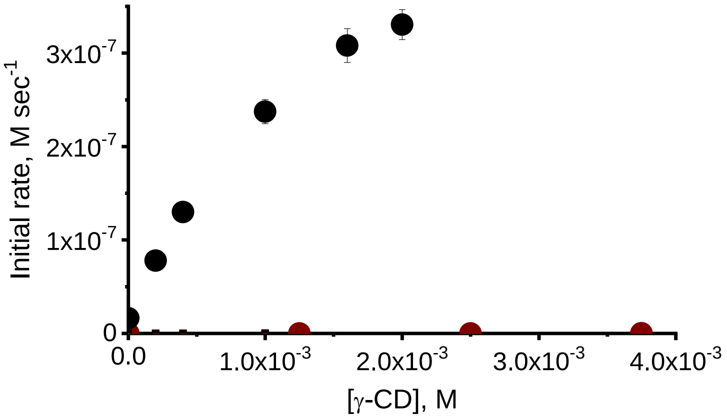


**Figure S11.** Initial rates of HPNP cleavage in the presence of increasing concentration of γ-CD (●), data obtained in the presence of **1**·Zn(II)$_2$ at 0.5 mM concentration (●, see Figure 1B) are reported for comparison. Conditions: pH = 8.0, [EPPS buffer] = 0.02 M, [HPNP] = 1.7 × 10$^{-4}$ M, 40 °C.

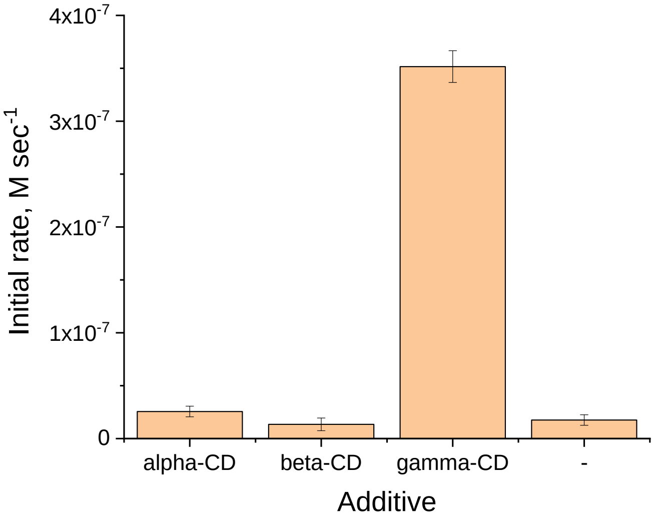


**Figure S12.** Initial rates of HPNP cleavage in the presence of **1**·Zn(II)$_2$ and with no or different cyclodextrins added. Conditions: [**1**·Zn(II)$_2$] = 0.5 mM, [CD] = 5 mM, pH = 8.0, [EPPS buffer] = 0.02 M, [HPNP] = 1.7 × 10$^{-4}$ M, 40 °C.

### 4.2. pH dependent kinetic experiments

The dependence of the reaction rate from pH was also investigated (Figure S10). We obtained a sigmodal profile in the rate vs pH plot. This behavior indicates that the reactive species undergo a single ionization with a $pK_a$ value of 8.16, which is consistent with the deprotonation of a water molecule bound to a TACN·Zn(II) unit.

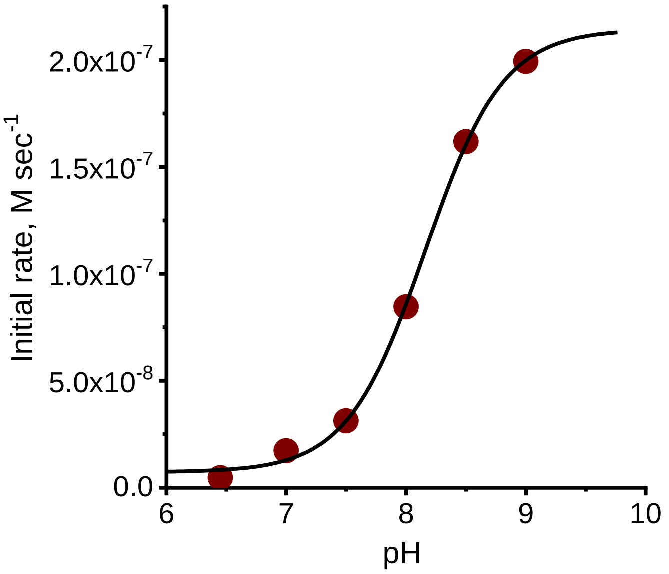


**Figure S13**. Initial rates of HPNP cleavage in the presence of $Zn_2$(**1**) and γ-cyclodextrin at different pH values. Conditions: [$Zn_2$(**1**)] = $1.5 \times 10^{-4}$ M, [γ-CD] = $1.5 \times 10^{-3}$ M, [buffer] = 0.02 M, [HPNP] = $5.0 \times 10^{-4}$ M, 40 °C in mQ. The line represents the best fitting of the data according to the equation reported in part 1. The $pK_a$ value obtained is 8.16.
